# Evidence for fluorescence-supported species recognition in syntopic harvestmen

**DOI:** 10.1038/s41598-026-36335-2

**Published:** 2026-01-21

**Authors:** Stefan Friedrich, Martina Schwager, Martin Heß, Frank Glaw, Tobias Lehmann

**Affiliations:** 1 Faculty of Biology, Biocenter LMU, Ludwig-Maximilians-University Munich, Großhaderner Str. 2, 82152 Planegg-Martinsried, Germany; 2 Department of Arthropoda varia, SNSB-Bavarian State Collection for Zoology, Münchhausenstr. 21, 81247 Munich, Germany; 3 Department of Applied Sciences and Mechatronics, Munich University of Applied Sciences, Lothstr. 34, 80335 Munich, Germany; 4 Department of Herpetology, SNSB-Bavarian State Collection for Zoology, Münchhausenstr. 21, 81247 Munich, Germany

**Keywords:** Evolutionary ecology, Animal behaviour

## Abstract

Biofluorescence is reported as a widespread phenomenon in a variety of animal species, but in most cases its biological relevance is poorly understood. Possible functions of biofluorescence as visual signals for species recognition are disputed or have not been convincingly demonstrated. Here, we report on the discovery of a strongly fluorescent structure in largely crepuscular and nocturnal harvestmen. Five syntopic species of harvestmen from the family Cosmetidae were observed and collected in a lowland rainforest of Peruvian Amazonia. Their most prominent species-specific character is a whitish structure on the dorsal scutum, called equuleus. Its shape is extremely constant intraspecifically, but strongly differs among these closely related species. The equuleus shows strong fluorescence in the blue frequency spectrum, which is enhanced by a subjacent layer of guanine crystals, acting as a reflector for the incoming light, as well as for the emitted fluorescence. We hypothesize that the equuleus most likely provides a visual signal for intra- and inter-specific species recognition in dim visible light, which is amplified by UV-induced fluorescence, excited by moonlight.

## Introduction

Ultraviolet (UV)-induced fluorescence is a widespread phenomenon across the animal kingdom. In recent years, numerous studies have investigated fluorescent structures in various taxa, including fish^1,2^, amphibians^3–5^, reptiles^6–9^, birds^10,11^, and mammals^12,13^.

Among terrestrial arthropods, scorpions are famous for their strong whole-body fluorescence and are one of the most extensively studied groups regarding the biological functions of fluorescence. Kloock^14^ examined the UV-related fluorescence in two sympatric scorpion species and found no significant difference in emitted wavelength between males and females and only minor interspecific variations (approximately 2 nm). These findings suggest that fluorescence may not serve as a mechanism for sex or species recognition^14^. In other studies^15,16^ fluorescence in scorpions is regarded as playing an active role in light detection, potentially helping them avoid moonlight exposure and locate cover. However, evidence remains inconclusive, and fluorescence in scorpions might simply be a by-product associated with the stiffening process of the cuticle^17^. UV-induced fluorescence has also been documented in various other terrestrial arthropods, including over 40 genera of spiders^18^, as well as harvestmen^19,20^, millipedes^20,21^, and insects^22^.

According to Marshall & Johnsen^23^, the only well-supported evidence of intraspecific communication through UV-induced fluorescence in terrestrial arthropods involves the jumping spider *Cosmophasis umbratica* Simon, 1903 provided by Lim et al.^24^. These authors demonstrated that this species uses sex-specific UV and fluorescence signals as visual cues for mate selection.

Kury and Barros^25^ erected the genus *Taito* within the neotropical harvestmen family Cosmetidae with description of eight new species from Amazonia. Several species within this genus coexist sympatrically, which is typical of Amazonian habitats. In their work, Kury and Barros introduced the term “equuleus” to describe a white or yellowish structure on the dorsal scutum that contrasts sharply with the surrounding dark brown coloration. The shape of the equuleus is the most prominent species-specific character beside spination of male leg IV and the structure of male genitalia^25,26^.

During fieldwork conducted at the biological field station and Área de Conservación Privada (ACP) Panguana in the evergreen lowland rainforest of Amazonian Peru, we observed and collected five syntopic species of cosmetid harvestmen. As described by Kury and Barros^25^, each species exhibited a distinct equuleus shape, enabling preliminary species identification in the field. Under UV light, all species displayed strong fluorescence in their equulei.

This raises intriguing questions: why does the shape of the equuleus remain so constant within each single species of cosmetid harvestmen without intersexual variation, but differs significantly between syntopic species? Moreover, why does this structure exhibit such an intense fluorescence? And: Are cosmetid harvestmen able to use these visual signals in intraspecific and interspecific interactions?

In this study, we aim to provide evidence that UV-induced fluorescence may serve as a communication mechanism in harvestmen for species recognition, a function previously unreported for these arachnids.

## Materials and methods

### Study area and specimen collection

During several field trips between 2013 and 2017 five cosmetid species were observed at and collected from South America, Amazonian Peru, Huánuco Department, Puerto Inca Province, Yuyapichis District, Área de Conservación Privada (ACP) Panguana, near Rio Yuyapichis, 09°37’ S, 74°56’ W (230–260 m a.s.l.). Specimens were collected by hand at night by using an UV torch and preserved in 75–96% ethanol.

### Photos

Photos in the field were taken with a DSLR. Photo series in the lab were taken with a NIKKOR 85 mm f/3.5 G lens mounted on a Nikon D7000, combined with a Cognisys STKS-C-StackShot apparatus. As UV light source a Tank 007 TK-737 (395 nm) torch was used.

### Semithin sections of the equuleus

Specimens preserved in 75% ethanol were further prepared for sectioning. After dissection of cuticula and underlying tissue of equuleus, specimens were dehydrated in a graded acetone series from 30% to 100% and embedded in Glycidether 100. The equuleus was cut semithin (1–2 μm), using a HistoJumbo diamond knife on a RMC-MTXL ultramicrotome. Sections were either stained with methylene blue after Richardson et al.^27^ or inspected unstained for UV fluorescence. Sections were studied with a LEICA DM RBE fluorescence microscope.

### Fluorescence spectroscopy

Fluorescence excitation and emission spectra were recorded with a Fluorolog (Horiba) fluorimeter. All fluorescence spectra were corrected for the wavelength-dependent output of the Xenon lamp. Intensity of fluorescence is given by the detected emission divided by the simultaneously measured intensity of the lamp at the same excitation wavelength. The data points with an increment of 1 nm and a bandpass of 5.5 nm were obtained by integrating the emitted photons over 0.1 s. Recorded spectra were as follows:

1. *Emission spectra* Fluorescence spectra were recorded from 405 to 560 nm and 433 to 560 nm with the excitation wavelengths 378 nm and 409 nm which represent the two excitation maxima. The shift of the emission range relative to the excitation range prevents an overexposure to the irradiation beam and ensures that the detected signal originates from the fluorescence.
2. *Excitation spectra* They were recorded for the emission at 458 nm and 479 nm (max. emission wavelengths) in dependence of the excitation wavelength from 290 to 430 nm.
3. *Excitation-emission matrix* Emission spectra were obtained as a function of the excitation wavelength. The excitation wavelength ranged from 290 to 430 nm. For each excitation wavelength varied by 1 nm, the corresponding emission spectrum was recorded. The emission wavelength region was adjusted to four excitation ranges (290–385 nm, 386–400 nm, 401–415 nm and 416–430 nm) in order to avoid detection of the exciting radiation in the emission spectrum.

### Histology of the eyes

Samples of *V. adrik* stored in 70% ethanol were rehydrated in a graded ethanol series and postfixed overnight in Karnovsky’s fixative (buffered 2.5% glutaraldehyde + 2% formaldehyde) at RT. Eyehills were dissected with fresh razor blades.

For light microscopy an eyehill was postfixed with 1% OsO_4_ on ice, dehydrated in a graded acetone series, infiltrated stepwise with Spurr’s low viscosity resin, oriented in flat silicone embedding molds and polymerized 2d at 60 °C. The resin block was trimmed and cut into a complete semithin series (250 horizontal planes @ 1 μm on a Science Services MT-7000 ultramicrotome with a DiATOME Histo Jumbo diamond knife), mounted in ribbons on glass slides, stained with Richardson’s reagent and sealed under cover slips with DePeX embedding medium. The ribbons were photographed using an Olympus BX61VS virtual slide acquisition system (objective UPlanS Apo 20x/0.75, camera CX10, software VS-ASW (Version 2.9.2, www.olympus-sis.com)). Subsequently images of single slices were extracted from the the*.vsi files manually using the display-2-clipboard function in OlyVIA software (Version 2.5, www.olympus-sis.com) (RGB tif files @ 1511 × 878 px, resolution reduced to 1.28 μm/px) and Adobe Photoshop CS6 (Version 13.0, www.adobe.com).

The image series was imported in Amira 3D-reconstruction software (Version 5.6, www.vsg3d.com) after removing single very thin or wedge shaped slices and cleaning from artifacts in the resin around the biological material. After manual alignment volume rendering (all structures) and surface rendering (eye cups) was performed.

Detailed light micrographs were taken on an Olympus CX41 microscope (objective UPlan S Apo 60x/1.2 W, camera DP25, software cellD (Version 2.6, www.olympus-sis.com). Due to slight unevenness of the 1 μm slices in the region of the pigmented eye cups, images were taken at several focal planes and combined to extended focal images with Combine ZP software (Version 1.0, www.combinezp.software.informer.com) (Fig. 4d).

For electron microscopy a second postfixation step with OsO_4_, potassium ferrocyanide, uranyl acetate and lead acetate followed the protocol of Hua et al.^28^. After dehydration in a graded acetone series the sample was embedded in hard-plus resin 812. The block was mounted on an aluminum stub with conductive glue, trimmed to a 1 mm^3^ cube, and covered with 20 nm gold using a Leica EM ACE200 sputter coater. Further sectioning and imaging was performed with a ThermoFisher Apreo VS block-face scanning electron microscope @ 1.78 kV, 50 pA, backscattering electron detector, scanning speed 3 µs/px, 16 bit grayscale images, 6144 × 4096 px). Overview images were taken with 67.4 nm/px, details with 33.7. Higher magnifications were not used due to the insufficient structural preservation (electron microscopic examination was not initially intended when collecting the samples). A rough series was cut from distal to proximal at a cutting plane distance of 10 μm to find an appropriate plane to count axon profiles in the optic nerve.

## Results

### Field observations

**Fig. 1:**
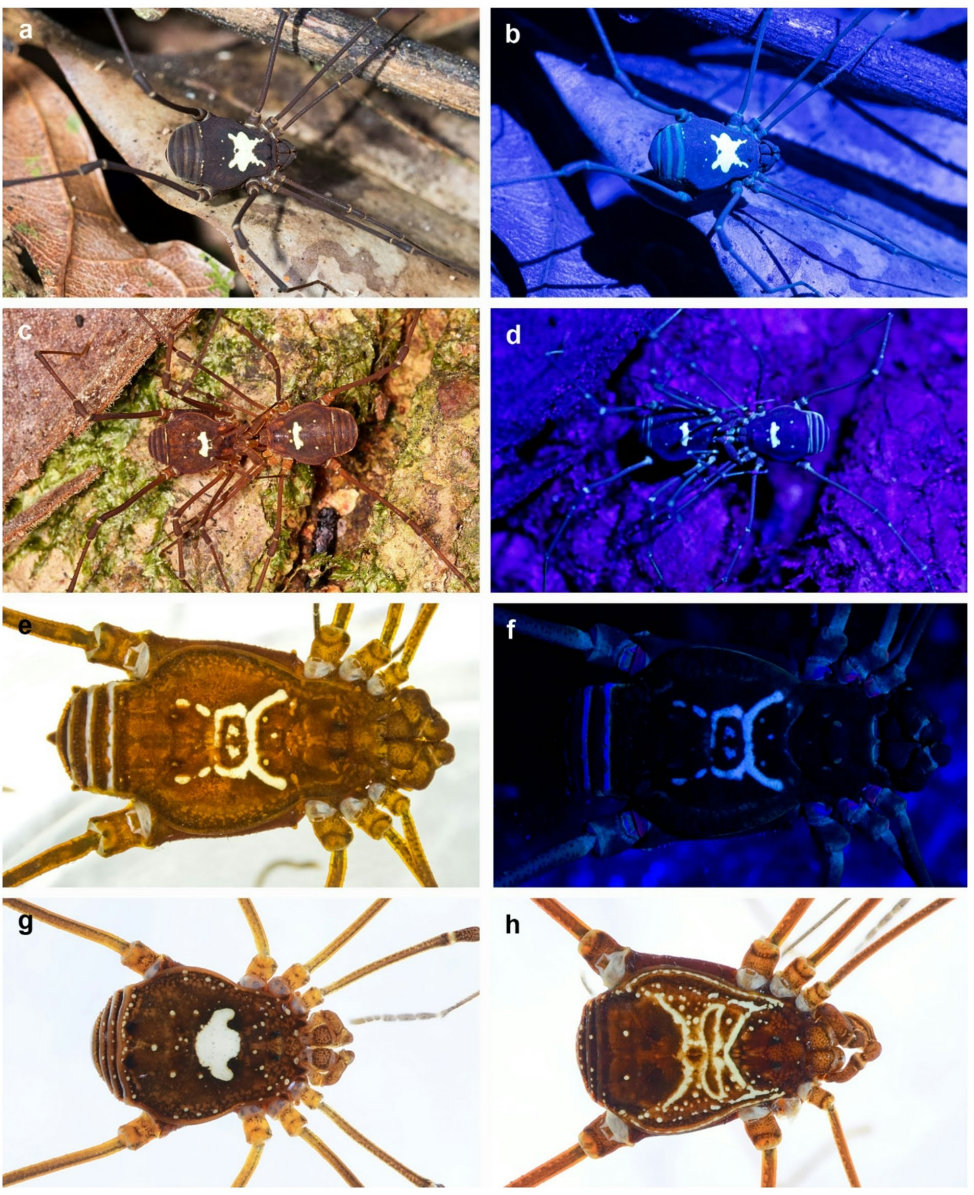
Five syntopic cosmetid harvestmen species observed in ACP Panguana, note species-specific fluorescent equuleus on the dorsal scutum. (**a**,**b**) *Vononana adrik* (Taitoinae) in the leaf litter on the forest floor at night under normal flashlight (**a**) and UV-light (395 nm) (**b**); (**c**,**d**) Mating Taitoinae Gen. sp. 2 under normal flashlight (**c**) and UV-light (395 nm) (**d**); (**e**,**f**) *Taito friedrichi* Kury, 2024 under normal flashlight (**e**) and UV-light (395 nm) (**f**); (**g**) Cosmetidae Gen. sp. 4. and (**h**) Cosmetidae Gen. sp. 5. under normal flashlight.

The five cosmetid species from ACP Panguana share the same habitat (the ground of the evergreen rainforest) at the same time syntopically for predation and mating in twilight after sunset and at night (Fig. 1c, d). All of them show preference for less densely forested areas, such as glades and pathways, and an increased mating activity around full moon phases.

All specimens were easy detectable due to a highly fluorescent structure on the dorsal side of the scutum, known as the equuleus. Although these species are similar in size, shape and body coloration, they differ clearly in the shape, size and pattern of the equuleus. This characteristic trait is remarkably consistent within each species^26^, but differs distinctly among the syntopic species^25^, with no differences between sexes within each species. Consequently, the species can be easily differentiated and identified in the field without further morphological examination.

### Morphology

Morphological analysis confirmed the presence of five species of the harvestmen family Cosmetidae at the ACP Panguana collection site. There exist several species-specific characteristics within this family, like the armature of leg IV and the structure of genitalia in males, features of the chelicerae and the anal operculum^25,26^. However, the most distinctive feature across all examined species is the shape of a structure on the dorsal scutum, referred to as the “equuleus”. In daylight, this white to yellowish-white structure contrasts sharply with the surrounding dark brown scutum (Fig. 1a,c,e,g,h). Under UV light, this contrast is even extremely enhanced, together with a strong fluorescence of the equuleus (Fig. 1b,d,f).

The species *Vononana adrik* (Friedrich & Lehmann, 2020) was the most frequently encountered cosmetid at this locality, alongside four other species. One of these additional species belongs to the closely related genus *Taito* (Kury & Barros, 2014) and has been recently described by Kury^29^ as *Taito friedrichi*. The remaining three Cosmetidae species from Panguana are so far undescribed species (Kury, pers. comm.).

### Histology of the equuleus

**Fig. 2:**
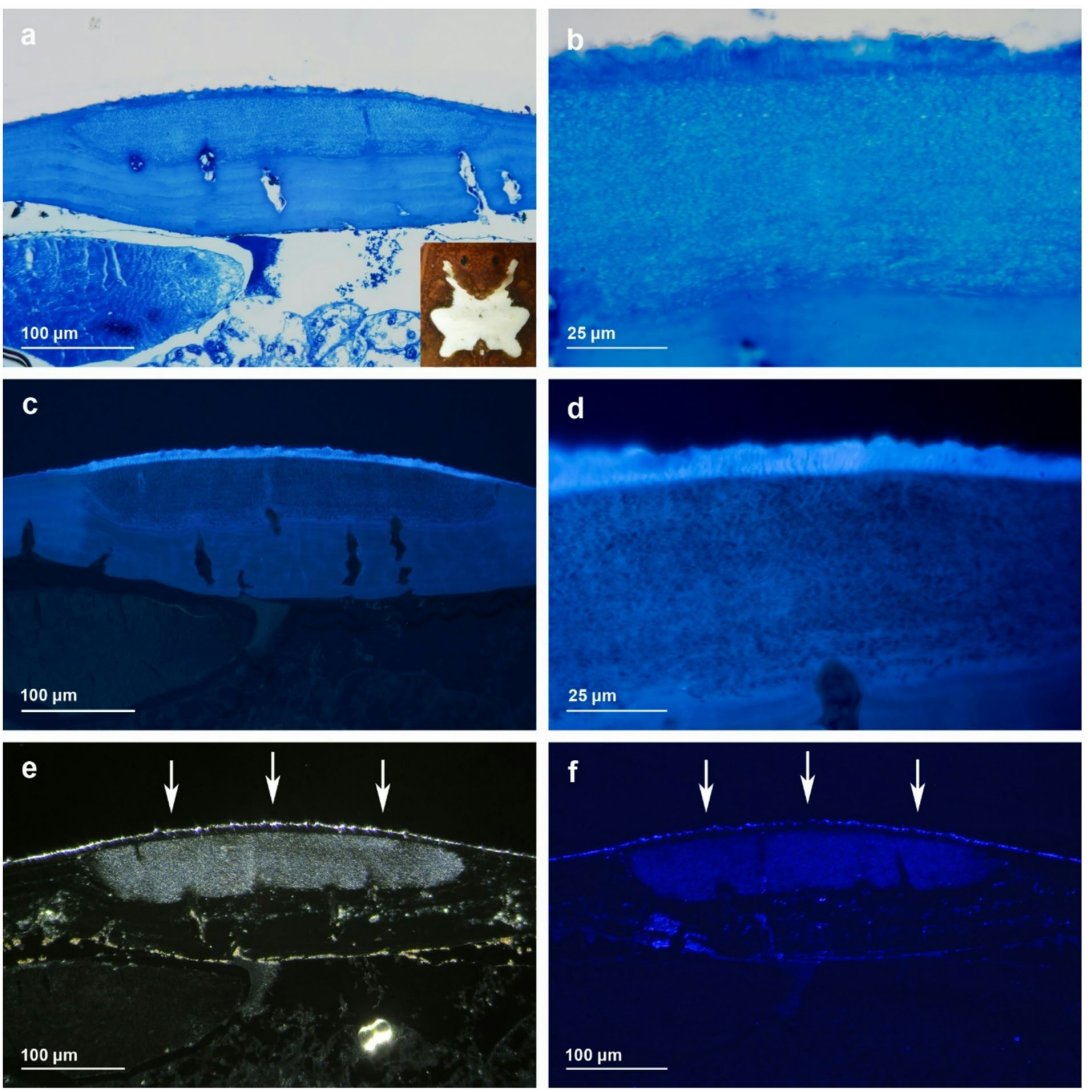
Histology of fluorescent equuleus of *Vononana adrik*. (**a**,**b**) transversal sections of equuleus (see inlay), light microscopic images. Region below fluorescent equuleus with layered guanine granules; (**c**,**d**) fluorescence microscopy shows that the cuticula is the fluorescent structure of the equuleus; (**e**,**f**) microscopic images without transmitting-light, arrows indicate direction of normal flashlight (**e**) and UV flashlight (**f**). Note layered structure below equuleus reflects incoming light.

Histology of the equuleus of *Vononana adrik* reveals that the cuticula is thickened in this region (approx. 12 μm) versus approx. 6 μm outside the equuleus (Fig. 2a,b). Exactly and exclusively below the cuticula of the equuleus, a structure of numerous parallel arranged reflecting crystal layers of 55 μm thickness is found (Fig. 2a,b). This multi-layered crystal structure is typical for guanine (C_5_H_5_N_5_O).

Fluorescence microscopy shows that the cuticula is the fluorescent structure of the equuleus (Fig. 2c,d). At the subjacent multi-layered structure, no or little fluorescence is recognizable (Fig. 2c,d). In Fig. 2e,f, the microscopic images are taken without transmitting light, but with lateral lighting. This setup simulates a vertical illumination of the equuleus and shows that the multi-layered structure below the equuleus is reflecting the incoming light, both the visible, as well as the UV light.

### Fluorescence photography and spectroscopy

**Fig. 3:**
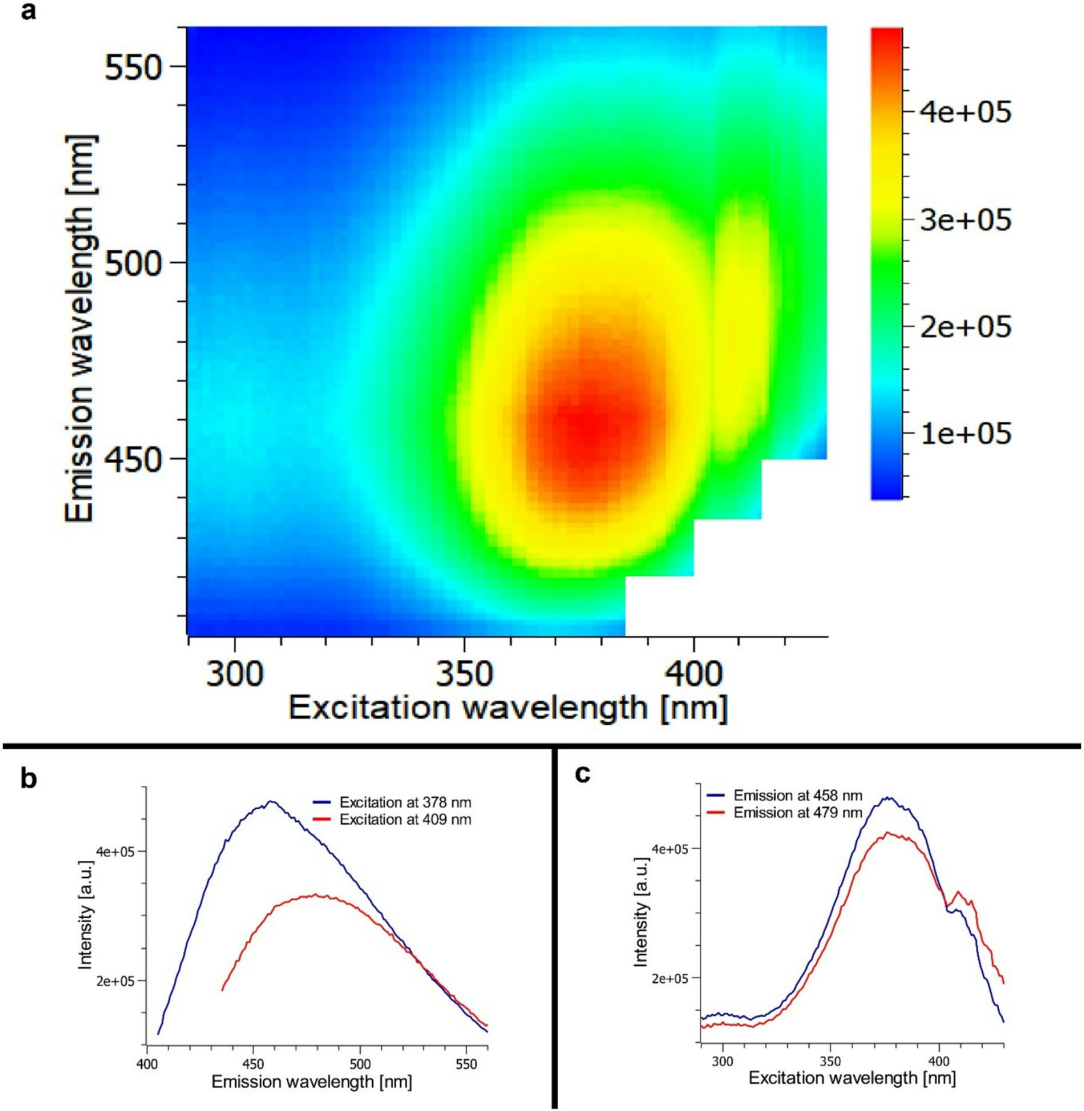
Fluorescence spectroscopy of the equuleus of *Vononana adrik*. (**a**) Excitation-emission matrix with emission range from 420 nm to 515 nm resulting in bright greenish blue fluorescence; (**b**) Emission spectra with the maximum at 458 nm upon excitation at 378 nm and around 479 nm upon excitation at 409 nm; (**c**) Excitation spectra for the emission at 458 nm and 479 nm with a maximum at 378 nm and a shoulder at 409 nm. [a.u.] = arbitrary unit.

The distinctive dorsal scutum pattern of *Vononana adrik* displays a bright greenish-blue fluorescence under UV irradiation, both in vivo and in ethanol-preserved specimens. A three-dimensional excitation-emission analysis reveals a broad emission band extending from 420 to 515 nm that produces this characteristic coloration (Fig. 3a). Fluorescence is excited within a wide UV range (350–415 nm), showing a primary excitation maximum at 378 nm and a secondary shoulder at 409 nm.

Two distinct excitation-emission pairs can be identified: excitation at 378 nm produces a strong emission peak at 458 nm, whereas excitation at 409 nm selectively induces a red-shifted emission maximum at 479 nm with an intensity amounting to 69% of the 458 nm peak (Fig. 3b). The gradual slope of the 378 nm-excited emission toward longer wavelengths indicates that the 479 nm component is always present, even under predominantly short-wavelength excitation.

Both emission peaks originate from the same extended excitation band (350–415 nm). Excitation at shorter wavelengths around 378 nm predominantly induces the 458 nm maximum, whereas excitation closer to 409 nm enhances the red-shifted 479 nm emission (Fig. 3c). The latter does not appreciably alter the overall greenish-blue appearance of the fluorescence, as it remains within the same spectral range as the dominant 458 nm peak. Instead, its primary effect is an increase in the integrated fluorescence intensity across the emission band. These excitation-emission relationships provide the basis for assessing how the spectral distribution of natural illumination, such as lunar light, may modulate the intensity of the observed fluorescence.

### Histology of the eyes

**Fig. 4:**
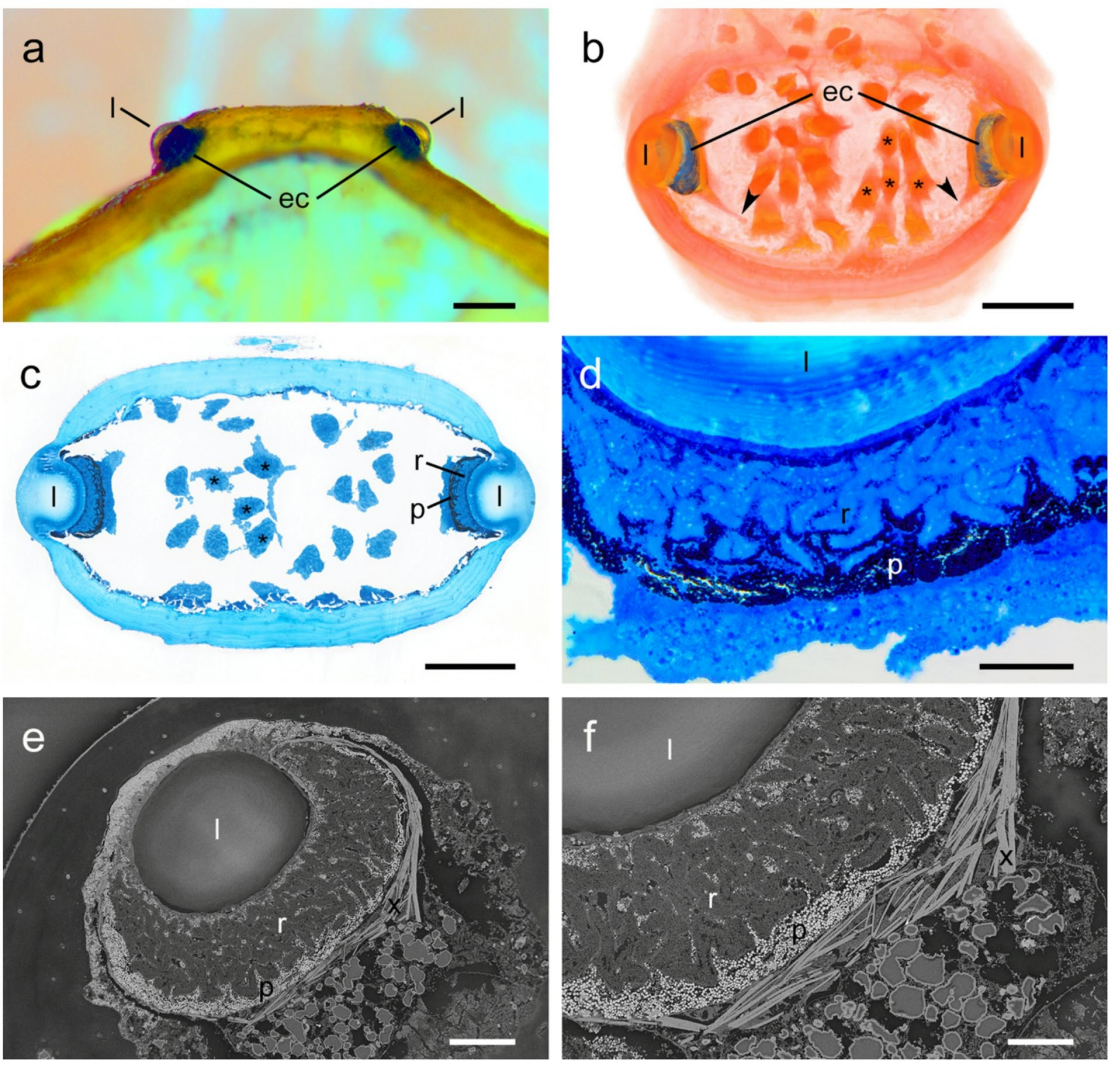
Eye structure of *Vononana adrik*. (**a**) Frontal view of the eyehill with two lense eyes, anterior portion of the prosoma removed with razor blade. Scale bar 250 μm; (**b**) Volume rendering of the eye hill + surface rendering of the eye cups based on a semithin section series. Scale bar 250 μm; (**c**) Semithin section through the eyehill at the level of the cuticular lenses (horizontal plane). Scale bar 200 μm; **(d)** Cross section of the eye cup, LM close-up. Scale bar 25 μm; **(e)** Oblique section through a lense eye, EM overview. Scale bar 50 μm; **(f)** Cross section of the eye cup, EM close-up. Scale bar 25 μm. **l** cuticular lens, **ec** pigmented eye cup, **p** pigment cells, **r** receptor cells, **x** crystalloid material, **„** eye nerve, * muscle fibre.

In the prosoma region *V. adrik* has a flat eye hill with two lens eyes. The optical axes point laterally at an angle of approximately 25° forwards and approximately 30° upwards. The cuticular lenses are clear, behind each of which a dark, pigmented eye cup shines through (Fig. 4a). In the specimen examined, the diameter of the roughly spherical lenses was approximately 155 μm. The retina or eye cup, made up of photoreceptor cells and pigment cells, lies against the inner side of the lens and forms an oval bowl distally with axes of approximately 155 × 105 μm and a depth of approximately 35 μm. The thickness of the retina is approximately 40–50 μm centrally, but forms a cone approximately 85 μm thick medio-ventrally, where the optic nerve leaves the eye cup obliquely backwards (Fig. 4b).

The photoreceptors are everted, lined by pigment cells, and surrounded by narrow processes containing pigment granules (Fig. 4c, d). Proximally, the optic cup is surrounded by crystalloid plates, possibly forming a guanine tapetum (Fig. 4e, f). Given their structural preservation, they can be described as cylindrical and most likely rhabdomerous; however, their orientation is irregularly bent in the specimens examined, and the cytoplasm is granularly degenerated (Fig. 4e, f). With a diameter of 7–8 μm, spacing of ca. 16.5 μm and a surface area of the cup of ca. 55 000 µm^2^ the total number of photoreceptors per eye can be estimated at ca. 190. The count of axon profiles in the optic nerve indicates 175–185 cells. For the given values of photoreceptor density and eye cup curvature, this results in a potential angular resolution of max. 6° under the assumption of a straight native orientation. This could be further reduced by underfocusing due to the small distance between the cuticular lens and the retina.

## Discussion

Harvestmen of the family Cosmetidae seem to be able to detect light at wavelengths in the greenish blue emission range of *Vononana adrik*’s equuleus, as well as in the near UV spectrum, which has been shown in the studies of Meyer-Rochow and Liddle^30,31^ for two cave inhabiting opilionid species from New Zealand. Both species, *Forsteropsalis tumida* (Forster, 1944) (Palpatores: Neopilionidae) and *Hendea myersi cavernicola* Forster, 1954 (Laniatores: Tryaenonychidae) are predating the bioluminescent glowworm *Arachnocampa luminosa* (Skuse, 1891) (Diptera: Kleropactidae), whose emitted light has a maximum at 487 nm^32^. In their studies, also artificial light sources of the same wavelength had the effect of attraction to the harvestmen. The wavelength of this glowworm’s emission is within the visible bright blue-green emission spectrum of *Vononana adrik*’s equuleus and is a strong indicator that these wavelengths can be recognised by the harvestmen. Moreover, in these studies the harvestmen were exposed to UV light (348 ± 20 nm), which caused a flight reaction, a normal behaviour for cavernicolous species to avoid the cave’s exit. This demonstrates also sensitivity to light in the UV range.

In contrast to spiders, harvestmen have only one pair of eyes, which is placed in the middle of the cephalothorax enabling an almost panoramic view. It could be argued that the position of their eyes might not allow them to see their own dorsal pattern and those of their conspecifics. However, given that free living harvestmen in the forest don’t move on a plane surface, but in a 3D space, caused by leaves, sticks and stones, we conclude that the syntopic cosmetids from Panguana are easily able to recognize visually the fluorescent structure on the other specimens’ dorsal scutum.

In the case of *Vononana adrik*, the multi-layered guanine structure directly below the equuleus seems to have an optical function as a mirror and reflector, amplifying the incoming light as well as the fluorescence from the cuticula (Fig. 2). Such multi-layered structures of rhombic guanine platelets are regularly found in the eyes of chelicerates also acting as an amplifying mirror in inverse retinas^33–36^. Furthermore, a similar design with reflecting guanine crystals is found in the fluorescent iridophores in the Namibian desert geckos^8^ and in iridocytes in the skin of fish^37^, as well as in eye structures of some reptiles (crocodiles and chameleons) and deep-sea fish^38^. Beside this, a lot of arachnids and some amphibians convert ammonia to guanine, to enable excretion with a minimal loss of water^37^. Summing up all biological functions of guanine, optical purposes seem to play a prominent role beside excretion.

Guanine’s standard fluorescence has a broad excitation range between 170 and 290 nm with four maxima, and emission maximum near 350 nm^39^. The fluorescence is largely independent of the excitation wavelength and exhibits a maximum intensity for an excitation at 280 nm with a full width of half maximum (FWHM) of around 80 nm and a Stokes shift of 70 nm. By contrast *V. adrik*’s excitation range is compared to guanine red shifted by about 98 nm (Fig. 3a, b). Similarly, the absorption characteristics of guanine show a strongly decreasing absorption at wavelengths above 300 nm, with less than 10% of the incident light being absorbed at 320 nm and a negligible amount of light being absorbed in the equulueus excitation region (Fig. 3c).

This indicates that the absorption and excitation regions of guanine are distinctly separate from those of the equuleus. Importantly, guanine does not absorb within the excitation range of the equuleus but acts as a reflective layer: incident excitation light that is not absorbed during the first passage through the photoactive cuticular layer is reflected by the guanine crystals and directed through the layer a second time. This repeated traversal markedly enhances the probability of absorption and thereby increases fluorescence yield. In addition, fluorescence photons, which are emitted isotropically, are partially reflected at the guanine layer, reducing losses by backscattering and further amplifying the apparent emission intensity. As our excitation of the semi-thin cuts of the equuleus with UV light clearly shows, the actual source of fluorescence is located in the cuticula, not in the subjacent guanine layer. Like in all other cases of cuticular fluorescence in arachnids (Scorpiones, Xiphosura, and Opiliones: Gonyleptidae), the fluorescent source is most probably a hyaline layer within the exocuticle, which appears to be a plesiomorphic trait in all chelicerates^19,40^.

*Vononana adrik*, like the other cosmetid harvestmen at ACP Panguana, is active during twilight and at night-time. Therefore, the ambient light inducing fluorescence primarily consist of direct or indirect moonlight and diffuse dawn light. The specimens inhabit the evergreen rainforest, where they are typically observed moving on twigs and leaves on the ground. Despite the dense foliage, direct light still reaches the forest undergrowth. In shaded areas, the spectrum of the moonlight (as reflected sunlight) shows an enhanced intensity in the blue region due to the frequency dependence of Rayleigh scattering^41^. The lunar light spectrum reaching these harvestmen varies seasonally and depends on the geographic position of the habitat. At the collection locality ACP Panguana (latitude 9°37’ S), the light has a steep incidence angle, allowing for a perpendicular moon position approximately every 14 days.

In tropical regions, the lunar spectrum passes through an air mass coefficient of about 1.0–1.1, resulting in relative intensities of ~ 28% in the UV range around 378 nm and ~ 51% in the blue range around 409 nm compared to the maximum at 473 nm. These values are slightly red-shifted relative to the solar spectrum^42–44^. The habitat of the specimens is further characterized by exceptionally high humidity, as tropical rainforests have some of the highest average annual water vapor contents in the lower atmosphere^45^. Increased water vapor is known to slightly enhance the blue spectral component compared to a clear day^46^, and under rainforest conditions such an effect can be expected. This would shift the natural illumination somewhat closer to the excitation range of harvestmen fluorescence.

The excitation characteristics of *Vononana adrik* include both shorter-wavelength UV light around 378 nm and longer-wavelength blue light around 409 nm. However, the lunar spectrum provides almost twice the relative irradiance in the 409 nm region compared to 378 nm (51% vs. 28%), and this effect is further strengthened by the humidity-enhanced blue component. Consequently, excitation in the 409 nm range contributes effectively to the overall excitation and leads to an increased fluorescence intensity. While the overall strength of the emission may vary with lunar phase, twilight conditions, weather, and microhabitat, the spectral composition of the emitted light remains essentially unchanged.

Fluorescence spectra are moreover highly conserved across arachnid lineages and typically confined to a greenish-blue emission range^17,19,47^, suggesting that spectral characteristics contribute little to the differentiation of closely related species. Interspecific distinctions are more likely associated with variation in dorsal patterning. Current evidence further suggests that arachnid fluorescence functions primarily to enhance the visibility of existing cuticular structures (e.g., surfaces or shields) rather than to provide distinct spectral information^17,47^. Within this framework, the bright greenish-blue fluorescence of *Vononana adrik* may increase the visual prominence of the dorsal scutum pattern against the moonlit background, thereby facilitating its perception by conspecifics.

**Fig. 5:**
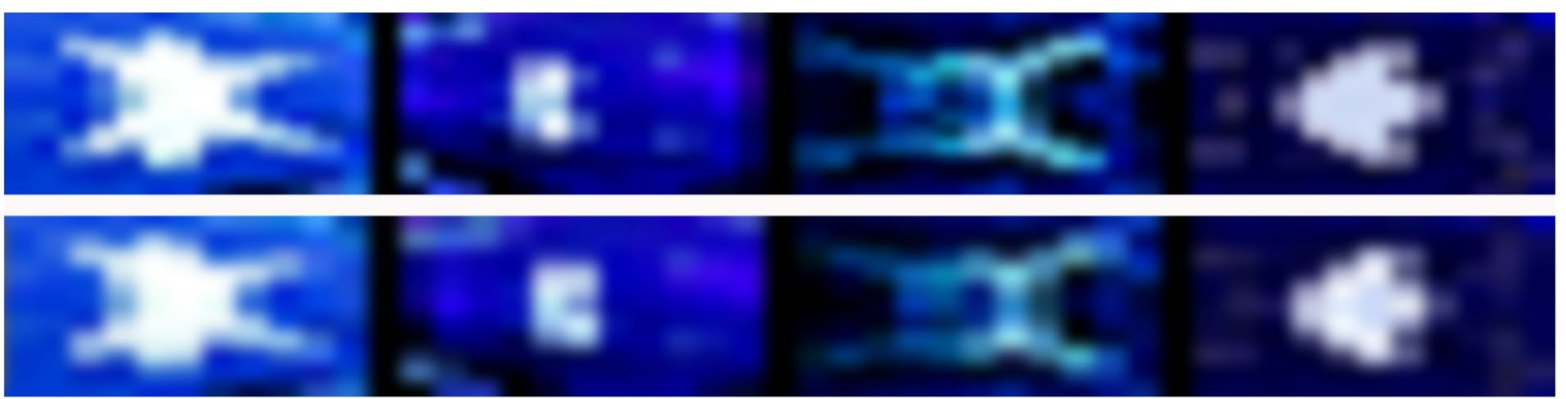
The simulations show how the equuleus patterns from Fig. 1b,d,f,g could be seen by a single eye of *V. adrik* at close range at an oblique angle and with some blurring due to underfocussing, under the assumptions mentioned above. Top row with 170 photoreceptors, bottom row with 120 photoreceptors or lower angular resolution.

The light and electron microscopic structural findings indicate fully functional eyes in the typical design of Opiliones: lenses, photoreceptors, pigment cells, optic nerve, etc^30,48^. Tapetal crystalloids on the back of the optic cups may indicate increased photosensitivity. The structural preservation does not allow for direct conclusions about the rhabdomeric structure of the photoreceptor cells (the sample was not fixed with the intention of later electron microscopic examination), but it does allow for many details of the cellular and fine-structure architecture of the eyes. Thus, the present study provides another example of the suitability of alcohol-preserved collection material for microscopic structural investigations^49^. The visual resolution of *V. adrik* can only be roughly estimated. Although we have no real evidence, we suspect a certain probability or at least possibility that the described dorsal patterns with their distinct contrast edges, which differ significantly between species, could be perceived in rough outlines and possibly distinguished in the close range (Fig. 5). If the actual resolution is insufficient to optically resolve the species-specific shape of the equuleus (which is somehow stabilized through evolution), then perhaps the overall size or brightness of the appearance may be sufficient. The presence of a tapetum suggests increased light sensitivity.

## Conclusions

Although biofluorescence is a widespread phenomenon, its biological significance remains unclear in most cases^22,23^, with only a few studies providing compelling evidence for its role in communication^24,50–52^. Recent papers^23,51,52^ emphasized that attributing specific functions to fluorescence requires exceptional evidence supported by a rigorous framework and identified five conditions suggestive of a function in communication^50^: A fluorescent compound (i) with known excitation and emission wavelengths (ii) is present in a visible body region and (iii) exhibited in a suitable light environment, (iv) producing a shift in coloration perceptible by potential viewers and (v) eliciting a behavioural response in the receiver.

Behavioural experiments, which were beyond the scope of this study, could be carried out in the future, since these harvestmen species are rather common at Panguana and easy to observe. Apart from this aspect we have shown that the studied harvestmen meet the other required criteria.

Our findings suggest that the cosmetid harvestman *Vononana adrik*, along with its syntopic relatives, demonstrates cuticular fluorescence in a species-specific structure as a means of inter- and intraspecific communication among these arthropods. As crepuscular and nocturnal species, these harvestmen seem to use UV-induced fluorescence to enhance the visibility of their distinctive species-specific feature under twilight conditions in order to distinguish between conspecifics and non-conspecifics. They might be able to detect both UV light and wavelengths of their own emitted fluorescence and the species-specific dorsal pattern are visible for conspecifics. At least the eye structure of *Vononana adrik* indicates a generally sufficient vision in terms of resolution and sensitivity.

Given that nature will probably not invest energy to establish sophisticated structures without any function (e.g., the guanine mirror), and based on the available evidence we believe that a function in communication is the most plausible hypothesis to explain the fluorescence of these harvestmen species.

## Data Availability

The data that support the findings of this study are available from the corresponding author upon reasonable request.
